# Reduced exercise capacity is associated with left ventricular systolic dysfunction in long‐term survivors of allogeneic hematopoietic stem‐cell transplantation

**DOI:** 10.1002/jcu.23264

**Published:** 2022-07-05

**Authors:** Richard John Massey, Ole Henrik Myrdal, Phoi Phoi Diep, Marta Maria Burman, Lorentz Brinch, Lars Lysgaard Gullestad, Ellen Ruud, Svend Aakhus, Jan Otto Beitnes

**Affiliations:** ^1^ Department of Cardiology Oslo University Hospital Oslo Norway; ^2^ Institute for Clinical Medicine Faculty of Medicine, University of Oslo Oslo Norway; ^3^ Department of Respiratory Medicine Oslo University Hospital Oslo Norway; ^4^ Department of Hematology and Oncology, Division of Pediatric and Adolescent Medicine Oslo University Hospital Oslo Norway; ^5^ Department of Pediatric Research, Division of Pediatric and Adolescent Medicine Oslo University Hospital Oslo Norway; ^6^ Department of Hematology Oslo University Hospital Oslo Norway; ^7^ KG Jebsen Center for Cardiac Research University of Oslo Oslo Norway; ^8^ Center for Heart Failure Research Oslo University Hospital Oslo Norway; ^9^ Department of Circulation and Imaging, Faculty of Medicine and Health Science Norwegian University of Science and Technology Trondheim Norway; ^10^ Clinic of Cardiology St. Olavs Hospital Trondheim Norway

**Keywords:** cardio‐pulmonary exercise test, echocardiography, global longitudinal strain, left ventricular ejection fraction, peak ventilatory oxygen‐uptake, three‐dimensional echocardiography

## Abstract

**Purpose:**

Exercise intolerance is a common complication in survivors of allogeneic hematopoietic stem‐cell transplantation (allo‐HSCT). The aim of this study was to determine if cardiac function measured with echocardiography is associated with exercise capacity measured with cardio‐pulmonary exercise tests in long‐term survivors treated in their youth with allo‐HSCT.

**Methods:**

The study included 96 patients, of which 54.2% were female, aged 34.9 ± 11.6 years and 17.7 ± 9.3 years after allo‐HSCT. Reduced exercise capacity was defined as <85% of predicted‐peak oxygen uptake (VO_2peak_). Linear regression was used in the prediction of VO_2peak_ (ml/kg/min). Receiver operating characteristic evaluated the accuracy of predicting reduced exercise capacity.

**Results:**

VO_2peak_ was 36.2 ± 7.7 ml/kg/min and 43 (44.8%) had reduced exercise capacity. Left ventricular ejection fraction was 55.4 ± 5.9% and global longitudinal strain (GLS) was −17.6% ± 2.0%. Left and right ventricular functions were significantly lower in survivors with reduced exercise capacity. Increased body mass index, lower physical activity score, reduced pulmonary function (by forced expiratory volume in 1‐s) and reduced left ventricular systolic function (by GLS) were significant independent predictors for reduced VO_2peak_. GLS was superior to other echocardiographical indices for identifying reduced exercise capacity (area under curve = 0.64, *p* = 0.014).

**Conclusions:**

Left ventricular systolic dysfunction measured by GLS is associated with reduced exercise capacity in long‐term allo‐HSCT survivors.

## Graphical abstract

Exercise intolerance is a common complication in long‐term survivors of allogeneic hematopoietic stem‐cell transplantation. In this study, we found reduced oxygen uptake (VO_2peak_) by cardio‐pulmonary exercise tests to be associated with left ventricular systolic dysfunction by echocardiography. In particular, global longitudinal strain was found to have superior ability to predict VO_2peak_ compared to other echocardiographical parameters.

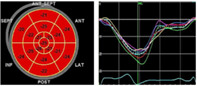

## 1 INTRODUCTION

Allogeneic hematopoietic stem‐cell transplantation (allo‐HSCT) is increasingly being selected as a potentially curative therapy for young recipients with malignant and non‐malignant disease.^1^ In parallel with advances in treatment, more recipients are surviving the initial years after transplantation.^2^ Consequently, increased focus has been directed towards improving quality of life by reducing therapy related complications.

A common complication in survivors of cancer therapies is exercise intolerance. The main limiting factors for exercise capacity are cardiac and pulmonary function, hematological capacity and metabolism in skeletal muscle. In long‐term survivors of allo‐HSCT, the risk of myocardial and pulmonary disease is elevated due to chemotherapy, high rates of cardiovascular risk factors, graft‐versus‐host disease (GVHD) and physical de‐conditioning. A challenge for the clinician is to identify cardiac dysfunction as the cause and to promptly initiate treatment to prevent irreversible heart failure. Cardio‐pulmonary exercise tests (CPET) is a widely used method to differentiate reasons for dyspnea. In addition, the acquirement of peak oxygen uptake (VO_2peak_) provides valuable prognostic information on cardiovascular related and all‐cause mortality.^3^, ^4^, ^5^

Echocardiography provides confirmation of cardiac dysfunction and insight into mechanisms for reduced exercise capacity. However, relationships between VO_2peak_ and left ventricular (LV) systolic function by echocardiography have been inconsistent. This is in part due to inadequacies with traditional measurements of left ventricular ejection fraction (LVEF). Three‐dimensional (3D) imaging and global longitudinal strain (GLS) from speckle tracking echocardiography (STE) have shown to improve sensitivity in detecting subtle effects of cardiotoxicity.^6^, ^7^, ^8^, ^9^, ^10^ As such, these methods may align better with observations of oxygen uptake. In particular, is the reported ability of GLS to predict functional capacity in patients with myocardial dysfunction.^11^, ^12^

Previous examinations in this cohort have shown a high prevalence of left ventricular systolic dysfunction (LVSD) that is strongly associated with first‐line anthracycline therapies.^13^ The present study aims to determine if cardiac function measured with echocardiography is associated with exercise capacity measured with CPET, in long‐term survivors of allo‐HSCT treated in childhood, as adolescents or young adults (CAYA). We hypothesize that modern techniques such as 3D‐LVEF or GLS are more accurate in determining relationships with the prognostic marker of VO_2peak_ (L/min/kg).

## 2 METHODS

### 2.1 Study design

This nationwide cross‐sectional study was designed to include all survivors of allo‐HSCT conducted at our institution in a multidisciplinary study investigating the long‐term effects of allo‐HSCT. Eligibility criteria were: Treatment at our national center for allo‐HSCT, age <30 years at transplantation, age >16 years at study inclusion and >5 years follow‐up time. Indications for allo‐HSCT were malignant and non‐malignant diseases. Survivors with Hurler syndrome were excluded due to the possibility of multi‐organ pathology as part of their primary disease. Written informed consent was obtained from all participants, and the study was approved by the Regional Committee for Medical and Health Research Ethics.

### 2.2 Clinical assessment

All participants underwent a medical examinations, questionnaires and blood sampling from June 2014 to February 2016. Dyspnea was classified according to the New York Heart Association (NYHA).^14^ Anthracycline cumulative dosage was converted to isotoxic doses of doxorubicin.^15^ Blood pressures were acquired after echocardiography (>30 min), in the supine position as the average of three measurements. Blood samples were collected after overnight fasting and analyzed at the hospital laboratory. N‐terminal pro‐brain‐type natriuretic peptide (NT‐proBNP) concentrations were determined by an electrochemiluminescence immunoassay (Roche Diagnostics, Basel, Switzerland). The lowest detectable level was 5n/L and manufacturer's recommendations were used for classifying elevated NT‐proBNP according to the age and sex specific cutoffs. Anemia was defined as reduced hemoglobin (males: <13.5 g/dL and females: <12 g/dL). Hypertension was defined as use of anti‐hypertensive drugs and/or systolic blood pressure (SBP) >140 mmHg or diastolic blood pressure (DBP) >90 mmHg. Hypercholesterolemia was defined as low‐density lipoprotein (LDL) >4.1 mmol/L (160 mg/dl) or use of lipid lowering medication. Diabetes mellitus was identified by hemoglobin HBA1c >6.5% (48 mmol/mol), fasting glucose ≥7.0 mmol/L or current use of glucose‐lowering medication. Obesity was classified as body mass index (BMI) ≥30 kg/m^2^. Acute graft‐versus‐host disease (aGVHD) was graded by Glucksberg scales and chronic GVHD (cGVHD) was graded by Shulman scales.^16^, ^17^

### 2.3 Physical activity

Physical activity was quantified from a self‐reported questionnaire (HUNT 2/3) that has been validated against measurements of VO_2peak_, METS calculations and international physical activity questionnaires in a comparative population.^18^ This calculated physical activity during a week as the product of weighted scores for the categories of frequency (scores of 0, 0.5, 1, 2.5, 5.0 ranging from ‘never’ to ‘almost every day’), intensity (scores of 1.0, 2.0, 3.0 ranging from ‘light exercise’ to ‘near‐exhaustion’) and duration (scores of 0.1, 0.38, 0.75, 1.0 ranging for ‘<15 min’ to ‘>60 min’).^18^ The range is 0 to 15 and higher values reflect greater weekly physical activity. An example: Exercising 2–3 times a week, for a total 60–180 min at a moderate intensity gives a physical activity score of 3.75. This scoring system generates a numerical scale that can be used to quantify the level of physical activity.

### 2.4 Pulmonary function

Spirometry was conducted as recommended by European Respiratory Society.^19^ Pulmonary function in this cohort has previously been described.^20^ For the present study, we used forced expiratory volume in 1‐s (FEV_1_) to represent lung function. The rational was two‐fold; FEV_1_ is readily attainable, and is strongly associated with bronchiolitis obliterans syndrome (BOS), which is the most clinically relevant respiratory disorder in this cohort. Percent of predicted‐FEV_1_ was calculated for each individual using recommended equations that adjust for race, ethnicity, sex and height.^21^ BOS was defined according to the National Institutes of Health (NIH) Consensus Criteria.^22^

### 2.5 Cardio‐pulmonary exercise test

CPET was conducted after echocardiography by experienced personnel at our institution. The test was performed on a treadmill (TechnoGym Runrace) using the modified Balke protocol.^23^ Incremental changes in ventilatory parameters were measured at regular intervals with Vyntus‐CPX (CareFusion). Predicted‐VO_2peak_ and percent of predicted‐VO_2peak_ were calculated for each individual based on equations that adjust VO_2peak_ by age and sex in a healthy control population.^24^ Reduced exercise capacity was defined as VO_2peak_ < 85% of predicted as recommended.^25^ Oxygen‐pulse was calculated by dividing VO_2peak_ by maximal heart rate, and predicted oxygen‐pulse by dividing percent of predicted‐VO_2peak_ by maximal heart rate.^25^

### 2.6 Echocardiography

Transthoracic echocardiograms were performed using Vivid‐E9 scanners, M5S‐D/M5Sc‐D (1.5–4.6 MHz) and 4 V‐D (1.5–4.0 MHz) probes and dedicated software (Echo‐PAC v113.1.3; GE‐Healthcare). The study followed current guidelines set by European Association of Cardiovascular Imaging and American Society of Echocardiography for the evaluation of LV and RV function, categorization of diastolic dysfunction and elevated filling pressures.^26^, ^27^, ^28^ Scanner settings were optimized and measurements were averaged from a minimum of three consecutive heart cycles. All examinations were conducted and analyzed by the same experienced investigator (R.J.M), en‐bloc and in random order after the last inclusion. After acquisition and prior to analyses, all echocardiograms were de‐identified and the investigator was blinded to medical treatments and CPET results. Parameters of systolic, diastolic and RV function were measured at separate occasions to reduce bias. Detailed technical description of echocardiographical methods, including variability tests and findings from analyses of LV and RV function have previously been published.^13^, ^29^ Measures of diastolic function included transmitral Doppler, myocardial tissue velocities (TVI), tricuspid regurgitation peak (TRP) and left atrium volume. Longitudinal strain of the RV by STE was calculated by two methods: RV‐global longitudinal strain (RV‐GLS) as the average value from six segments including free‐wall and septum, and RV free‐wall strain (RVFWS) as the average of the three free‐wall segments. Left ventricular systolic dysfunction (LVSD) was defined as reduced 2D‐LVEF (male: <52% and female: <54%) and/or GLS ≥−17%.^13^ Right ventricular systolic dysfunction (RVSD) was defined as reduction of at least two of the parameters: Fractional area change (FAC) <35%, tricuspid annular plane systolic excursion (TAPSE) <17 mm, RV systolic myocardial velocity (RV‐s') <9.5 cm/s, RV free‐wall strain (RVFWS) >−20%, and RV index of myocardial performance (RIMP) >0.54.^26^ Pulmonary artery systolic pressure (PASP) was calculated from TRP using the Bernoulli equation plus right atrium pressure estimated by size and respiratory variation of the inferior vena cava.^30^

### 2.7 Statistical analysis

Statistical analysis was conducted with SPSS version‐25 (SPSS, Inc.) and *p* < 0.05 was considered significant. Histograms and Shapiro–Wilk test were used to assess normality. Continuous data are reported as mean ± standard deviation or as median (25th, 75th percentile), and categorical data as numbers and percentages. Student's *t* test, One‐way analysis of covariance (ANCOVA) and Mann Whitney *U* were used to compare continuous data. Chi‐square and Fisher's exact test were used for comparisons of categorical data.

Cardiac function was compared in participants with normal (VO_2peak_ > 85% of predicted) verse reduced (VO_2peak_ < 85% of predicted) exercise capacity. ANCOVA was used to control for potential confounders to cardiac function created by this sub‐grouping. Covariates included age at examination, BMI, heart rate (HR) and SBP. An additional supplementary analysis with ANCOVA, Kruskal–Wallis test and with Bonferroni correction was used to compare cardiac function between participants with mildly reduced (75%–85% of predicted‐VO_2peak_), moderately reduced (<75% of predicted‐VO_2peak_) and normal exercise capacity (>85% of predicted‐VO_2peak_) (Table S1).

Pearson bivariate correlations determined the presence of linear relationships. Univariable and multivariable linear regressions were used to determine significant explanatory variables for VO_2peak_ (ml/kg/min). The multivariable analysis included a priori selected variables considered as central determinants for exercise capacity, and /or variables with *p* < 0.2 in the univariable analysis. All continuous variables were standardized and values presented with beta, confidence intervals and *p* value. Assumption testing included histograms, residual plots and assessment of multi‐collinearity by Pearson correlations, tolerance and variance inflation factor (VIF). Considerations were made to avoid over‐fitting. Pairwise omission was chosen to handle missing data. Receiver operating characteristics (ROC) were performed and areas under the curve (AUC) calculated to test the parameter's ability to identify patients with reduced exercise capacity.

## 3 RESULTS

**FIGURE 1:**
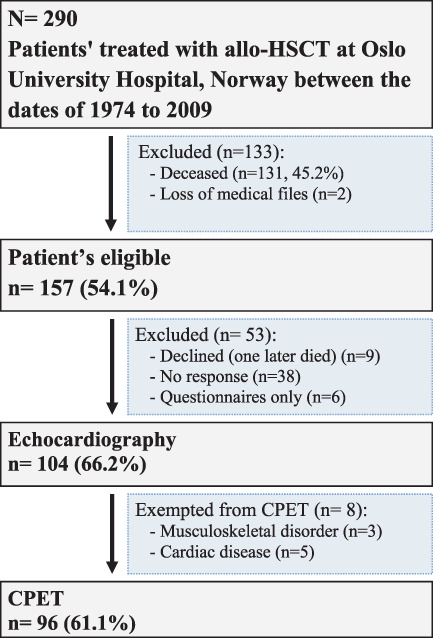
Flow chart of patient inclusion.

Two‐hundred and ninety patients received allo‐HSCT in the time frame specified for this study. Of these, 131 (45.2%) died prior to study start and two were excluded due to incomplete patient files (Figure 1). One‐hundred and fifty‐seven were eligible for inclusion, of which 104 (66.2%) were examined with echocardiography, spirometry and blood tests, and 96 (61.1%) examined with CPET. Eight participants were excluded from with CPET due to musculoskeletal disorders (*n* = 3), significant systemic hypertension and reduced cardiac function (*n* = 2), suspected coronary artery disease or abnormalities (*n* = 2) and congenital aortic stenosis (*n* = 1).

### 3.1 Patient characteristics

Table 1 shows patient characteristics and treatments. In the 96 survivors who completed both echocardiography and CPET: 52 (54.2%) were female, 34.9 ± 11.6 years of age at examination and with a follow‐up time of 17.7 ± 9.3 years. Malignant diseases were the most common (74.0%, of which 56.3% were female) indications for allo‐HSCT. In patients with malignant diseases, 43 (44.8%, 60.5% were female) received first‐line therapies with anthracyclines and two (2.1%) were treated with mediastinal radiotherapy. The median isotoxic cumulative anthracycline dosage was 270 mg/m^2^ and values ranged from 45 to 585 mg/m^2^. The majority received standardized myeloablative conditioning regimes consisting of busulfan and cyclophosphamide. Seven survivors had mild anemia (hemoglobin >10.9 g/dL). Thirteen (13.5%) were obese (≥30 kg/m^2^). Hypertension was found in 37 (38.5%) and 30 (31.3%) used anti‐hypertensive medication.

**TABLE 1:** Survivor characteristics

Variable	All survivors (*n* = 96)
Age at allo‐HSCT (years)	17.7 ± 9.3
Years to follow‐up (years)	17.2 ± 5.6
Age at examination (years)	34.9 ± 11.6
Female gender	52 (54.2)
Body mass index (BMI) (kg/m^2^)^a^	24.2 ± 5.1
BMI ≤18 kg/m^2^	8 (8.3)
BMI ≥30 kg/m^2^	13 (13.5)
Systolic blood pressure (mm Hg)^a^	121 ± 17
Diastolic blood pressure (mm Hg)^a^	71 ± 12
Heart rate (bpm)^a^	68 ± 11
Malignant/non‐malignant disease	71 (74.0) / 25 (26.0)
Mediastinal radiotherapy	2 (2.1)
Anthracyclines	43 (44.8)
Cum. Anthracycline dosage (mg/m^2^)	270 (140, 435)
Dosage >300 mg/m^2^	20 (20.8)
Myeloablative conditioning:	94 (97.9)
Chemotherapy (Bu/Cy)	88 (91.7)
Chemotherapy + TBI	6 (6.3)
No conditioning required	2 (2.1)
Graft‐versus‐host disease (GVHD)	62 (64.6)
Acute GVHD	25 (26.0)
Chronic GVHD	11 (11.5)
Both	26 (27.1)
New York Heart Association (NYHA)	
Class‐I	71 (74.0)
Class‐II	15 (15.6)
Class‐III	10 (10.4)
Class‐IV	0 (0)
Physical activity	
Physical activity score	3.8 (1.5, 5.0)
Risk factors	
Hypertension	37 (38.5)
Diabetes mellitus	2 (2.1)
Hypothyroidism	8 (8.3)
Hypercholesterolemia	13 (13.7)^b^
Smoking	26 (27.1)^b^
Current	10 (10.4)
Previous	16 (16.7)
Anemia^b^	7 (7.3)
Bronchiolitis obliterans syndrome (BOS)	10 (10.4)
Left ventricular systolic dysfunction (LVSD)	42 (43.8)
Laboratory parameters	
NT‐proBNP (ng/L)	48 (22, 83)
Elevated NT‐proBNP^c^	14 (14.6)
High‐density lipoprotein (mmol/L)	1.5 ± 0.4
Low‐density lipoprotein (mmol/L)	3.0 ± 0.8^b^
Hemoglobin (g/ml)^d^	14.3 ± 1.3
P‐CK‐MB (μg/L)	1.5 (1.1, 2.4)
Glomerular filtration rate (<60 ml/min/1.73 m^2^)	5 (5.2)
Creatinine (μmol/L)	76 (68, 89)
C‐reactive protein (mg/l)^e^	1.6 (0.6, 3.1)

*Note*: Data presented as mean ± SD, median (25th, 75th percentiles) or number (%).

Abbreviations: NT‐proBNP, N‐terminal pro‐brain‐type natriuretic peptide; P‐CK‐MB, plasma‐creatine kinase‐myocardial band; TBI, total body irradiation.

^a^ Measured at echocardiography.

^b^ *n* = 95.

^c^ Elevated NT‐proBNP = age 18–44 years male >86 ng/L, female 18–44 ng/L > 130 ng/L; age 45–54 years male >121 ng/L, female >249 ng/L.

^d^ Hemoglobin in males <13.5 g/dL and females <12 g/dL.

^e^ CRP lowest recordable value was 0.6 mg/L.

### 3.2 Pulmonary function

FEV_1_ was 3.27 ± 0.81 L/s, and percent of predicted‐FEV_1_ was 88.4% ± 17.8%. Percent of predicted‐FEV_1_ was significantly lower in survivors with BOS (56.0% ± 15.2% vs. 92.1% ± 13.9%, *p* < 0.001), anthracycline exposure (83.3% ± 18.2% vs. 92.6 ± 16.5%, *p* = 0.010) and LVSD (83.6% ± 20.1% vs. 92.2% ± 14.9%, *p* = 0.018). BOS was diagnosed in 10 (10.4%), of which five were female, eight (80%, *p* = 0.012) had cGVHD and seven had co‐existing LVSD (70%, *p* = 0.098).

### 3.3 CPET and exercise capacity

The main findings from CPET are summarized in Table 2. All individuals were exercised to peak effort: Borg scale ≥18 and/or respiratory exchange ratio (RER) ≥1.10. VO_2peak_ was significantly higher in males (male: 39.5 ± 6.7 ml/kg/min vs. female: 33.4 ± 7.5 ml/kg/min, *p* < 0.001). Percent of predicted‐VO_2peak_ was similar between sexes (male: 85.5% ± 13.2% vs. female: 91.5% ± 21.1%, *p* = 0.096), although significantly lower in survivors who were obese (78.4% ± 13.5% vs. 90.4% ± 18.2%, *p* = 0.026), had BOS (76.5% ± 15.0% vs. 90.2% ± 18.0%, *p* = 0.023) and treated with anthracyclines (84.7% ± 15.6% vs. 92.0% ± 19.4%, *p* = 0.049).

**TABLE 2:** Summary of CPET results for allo‐HSCT survivors (*n* = 96)

Variable	Value (*n* = 96)
Peak heart rate (bpm)	181 ± 15
Oxygen‐pulse (ml/beat)	14.1 ± 3.8
Percent of predicted oxygen‐pulse (%)^a^	88.1 ± 19.7
<85% predicted oxygen‐pulse (%)	44 (45.8%)
VO_2peak_ (l/min)	2.6 ± 6.7
VO_2peak_ (ml/kg/min)	36.2 ± 7.7
VO_2peak_ < 40 ml/kg/min	69 (66.3%)
VO_2peak_ < 30 ml/kg/min	25 (26.0%)
Percentage of predicted‐VO_2peak_ (%)^a^	88.8 ± 18.1
<85% of predicted‐VO_2peak_ (%)	43 (44.8%)

*Note*: Data presented as number (%), mean ± SD.

^a^ Values adjusted by age and sex.

Forty‐three (45.8%) survivors had reduced exercise capacity (VO_2peak_ < 85% of predicted). Collectively, survivors with reduced exercise capacity differed from survivors with normal exercise capacity by younger age (31.2 ± 11.8 years vs. 37.9 ± 10.6 years, *p* = 0.005), shorter follow‐up time (15.2 ± 5.9 years vs. 18.8 ± 4.7 years, *p* = 0.001), sex (male: 55.8% vs. female: 44.2%, *p* = 0.077) and larger BMI (25.4 ± 6.1 kg/m^2^ vs. 23.3 ± 3.9 kg/m^2^, *p* = 0.052). In addition, a higher prevalence of BOS (18.6% vs. 3.8%, *p* = 0.039), LVSD (55.8% vs. 34.0%, *p* = 0.032), higher frequency of dyspnea (NYHA ≥II, 41.9% vs. 13.2%, *p* = 0.001) and trend towards lower levels of physical activity (median scores: 2.0 vs. 3.8, *p* = 0.056) were found in the group with reduced exercise capacity. Oxygen‐pulse was significantly higher in males (male: 16.3 ± 4.0 ml/beat vs. female: 12.3 ± 2.4 ml/beat, *p* < 0.001), although percent of predicted equivalents of oxygen‐pulse did not significantly differ (male: 84.1% ± 19.8% vs. female: 91.5% ± 19.1%, *p* = 0.065). Forty‐four (44.8%) survivors had reduced oxygen‐pulse (<85% of predicted oxygen‐pulse).

### 3.4 Echocardiography and cardiac function

Good image quality allowed high reproducibility.^13^, ^29^ 2D‐LVEF was 55.4% ± 5.9% (range: 35% to 69%) and GLS was −17.6% ± 2.0% (range: −11.3% to −20.8%). LVSD was present in 42 (43.8%). Twelve (12.5% in total) had symptomatic LVSD (NYHA ≥II), of which 11 also had reduced exercise capacity. Elevated filling pressures were categorized in 21 (21.9% of total) with LVSD. RVSD occurred in 13 (11.5% of total, 11 had co‐existing LVSD) and diastolic dysfunction in the absence of LVSD was present in six (6.3%). Valvular heart disease was rare and no lesions were severe. Estimated PASP was less than 37 mmHg for all individuals.

Tables 3 and S1 shows cardiac function by echocardiography in all participants and in the sub‐groups with normal and reduced exercise capacity. The greatest levels of cardiac dysfunction corresponded to lowest levels of absolute or predicted values of oxygen uptake. Left‐ and right ventricular function remained significantly reduced after controlling for possible confounders. Significant group differences for LV systolic function remained after the removal of individuals with BOS (seven with co‐existing LVSD). Survivors with LVSD had lower values of percent of predicted‐VO_2peak_ (LVSD: 86.1% ± 18.5% vs. non‐LVSD: 90.8% ± 17.7%, *p* = 0.207) and predicted oxygen‐pulse (LVSD: 83.9 ± 15.3% vs. non‐LVSD: 91.4% ± 22.1%, *p* = 0.050). A diagnosis of LVSD increased the likelihood of reduced VO_2peak_ (OR 2.5, 95% CI 1.0 to 5.2, *p* = 0.032). Survivors with 2D‐LVEF <50% had lower percent of predicted‐VO_2peak_ (80.0% ± 11.7% vs. 90.2% ± 18.7%, *p* = 0.050) and trend towards reduced percent of predicted oxygen‐pulse (79.3% ± 14.0% vs. 89.6% ± 20.2%, *p* = 0.071).

**TABLE 3:** Cardiac function by echocardiography in survivors with normal (VO_2peak_ > 85% of predicted) and reduced exercise capacity (VO_2peak_ < 85% of predicted)

Variable	All survivors	Normal exercise capacity	Reduced exercise capacity	*p* value^a^	Adjusted *p* value^b^
Number	96	53 (55.2)	43 (44.8)	–	–
VO_2peak_ (ml/kg/min) (range)	36.2 ± 7.7 (19–54)	39.4 ± 6.6 (28–54)	32.3 ± 7.2 (19–44)	**<0.001**	–
Peak heart rate (bpm)	181 ± 15	179 ± 15	183 ± 15	**0.198**	–
Oxygen‐pulse (ml/beat)	14.1 ± 3.8	15.1 ± 4.1	12.9 ± 2.9	**0.003**	–
Predicted oxygen‐pulse (%)	88.1 ± 19.7	96.4 ± 17.9	77.9 ± 16.8	**<0.001**	–
Anthracycline exposure	43 (44.8)	21 (39.2)	22 (51.2)	0.258	–
Anthracycline dosage (mg/m^2^)	0 (0, 219)	0 (0, 207)	45 (0, 219)	0.477	–
NT‐proBNP (ng/l)	48 (22, 83)	47 (27, 82)	50 (18, 93)	0.583	–
NYHA (grade ≥ II)	25 (26.0)	7 (13.2)	18 (41.9)	**0.001**	–
Systolic function					
Cardiac output (l/min)	4.77 ± 1.13 (*n* = 95)	4.70 ± 1.16 (*n* = 52)	4.86 ± 1.11	0.502	0.140
2D‐LVEF (%)	55.4 ± 5.9	56.3 ± 5.5	54.3 ± 6.3	0.095	0.052
3D‐LVEF (%)	54.1 ± 4.9 (*n* = 83)	55.0 ± 4.4 (*n* = 49)	52.9 ± 5.4 (*n* = 34)	0.057	**0.046**
GLS (%)	−17.6 ± 2.0 (*n* = 94)	−18.0 ± 1.9 (*n* = 52)	−17.1 ± 2.0	**0.022**	**0.043**
MAPSE (mm)	13.1 ± 2.0	13.2 ± 2.1	12.9 ± 1.9	0.373	0.124
LV‐s' (cm/s)	8.1 ± 1.7	8.0 ± 1.8	8.3 ± 1.5	0.357	0.605
Diastolic function					
MV_DT_ (ms)	160 ± 39	160 ± 33	160 ± 46	0.949	0.199
MV_E/A_ ratio	1.6 ± 0.8	1.7 ± 0.9	1.6 ± 0.7	0.671	0.387
*e*' (cm/s)	11.1 ± 3.1	11.0 ± 3.2	11.2 ± 3.0	0.695	0.299
*E*/*e*'	6.4 ± 2.1	6.5 ± 2.4	6.4 ± 1.7	0.870	0.867
Right ventricular function					
FAC (%)	41.1 ± 5.3	40.9 ± 5.0	41.5 ± 5.6	0.576	0.759
RVFWS (%)	−27.0 ± 4.4 (*n* = 89)	−27.4 ± 4.5 (n = 51)	−26.4 ± 4.1 (*n* = 38)	0.319	**0.041**
RV‐GLS (%)	−21.8 ± 3.2 (*n* = 89)	−22.1 ± 3.3 (*n* = 51)	−21.4 ± 3.1 (*n* = 38)	0.268	**0.021**
TAPSE (mm)	20.8 ± 3.7	20.9 ± 3.8	20.7 ± 3.6	0.876	0.734
RV‐s' (cm/s)	11.1 ± 2.2 (*n* = 95)	10.9 ± 2.2 (*n* = 52)	11.3 ± 2.3	0.392	0.633
TRP (mmHg)	17.7 ± 3.4 (*n* = 72)	17.8 ± 3.6 (*n* = 41)	17.6 ± 3.1 (*n* = 31)	0.749	0.822

*Note*: Values presented as number (%), mean ± SD (and range) or median (25th, 75th percentiles). Significant *p* values (<0.05) are in boldface.

Abbreviations: *e*', Mean myocardial early‐diastolic velocity; *E*/*e*', MV_E_:*e*' ratio; FAC, fractional area shortening; GLS, global longitudinal strain; LV, left ventricular; LVEF, LV ejection fraction; LV‐s', LV systolic myocardial velocity (average of septum and lateral annulus); MAPSE, mitral annular plane systolic excursion (average of septum and lateral annulus); MV, mitral valve; MV_DT_, MV deceleration‐time; MV_E/A,_ ratio of MV early‐diastolic wave velocity (MV_E_) to MV late‐diastolic wave velocity (MV_A_); NT‐ProBNP, N‐terminal pro‐b‐type natriuretic peptide; RV, right ventricular; RVFWS, RV‐free‐wall strain; RV‐GLS, RV‐global longitudinal strain; RV‐s', RV systolic velocity (average of septum and lateral annulus); TAPSE: tricuspid annulus plane systolic excursion; TRP, tricuspid regurgitation pressure.

^**a**^ Comparison between survivors with normal and reduced exercise capacity made with Student's *t* test, Mann–Whitney *U* test, Chi‐square and Fisher's exact test.

^b^ ANCOVA with covariates of age at examination, BMI, heart rate at echocardiography and systolic blood pressure at echocardiography.

### 3.5 Predictors of VO_2peak_

VO_2peak_ (ml/kg/min) significantly correlated with BMI (*R* = −0.45, *p* < 0.001), oxygen‐pulse (*R* = 0.43, *p* < 0.001), mean myocardial early‐diastolic velocity (LV‐e', *R* = 0.37, *p* < 0.001), FEV_1_ (*R* = 0.35, *p* < 0.001), physical activity score (*R* = 0.32, *p* < 0.001), age at examination (*R* = −0.32, *p* = 0.002), transmitral deceleration‐time (MV_DT_, *R* = −0.31, *p* = 0.002), ratio of early and late transmitral velocity (MV_E/A_, *R* = 0.27, *p* = 0.007) and ratio of early diastolic filling velocity and *e*' (*E*/*e*', *R* = 0.25, *p* = 0.016). Regression analysis for the prediction of VO_2peak_ is presented in Table 4. Assumptions for regression were satisfied: Pearson's correlations <0.5, tolerance >0.6 and VIF <1.5 indicating limited risk of multi‐collinearity. Anthracyclines were negatively correlated with both cardiac and pulmonary function and hence not included in the multivariable model. *E*/*e*' was considered the most representative parameter for elevated filling pressures. Permutations with different indices of LV systolic and RV function were tested. LV systolic function represented by GLS (*β* = −0.18, *p* = 0.030), but not 3D‐LVEF (*β* = 0.14, *p* = 0.122) or 2D‐LVEF (*β* = 0.09, *p* = 0.305) was found to predict VO_2peak_. Unadjusted FEV_1_ and BOS as a categorical variable (also significant) were tested separately to control for effects of pulmonary diseases. In the final prediction model, the variables of sex, BMI, physical activity score, GLS and FEV_1_ were found to be significant independent predictors for VO_2peak_.

**TABLE 4:** Linear regression for the prediction of VO_2peak_ (ml/kg/min)

	Univariable			Multivariable *R* = 0.752 *r* ^2^ = 0.565		
Variable	*β*	95% CI	*p* value	*β*	95% CI	*p* value
Gender (female)	0.79	0.41–1.16	**<0.001**	0.69	0.34–1.04	**<0.001**
Age at examination (years)	−0.32	−0.51 to −0.12	**0.002**	−0.07	−0.23 to 0.10	0.427
Body mass index (kg/m^2^)	−0.45	−0.64 to −0.27	**<0.001**	−0.49	−0.66 to −0.32	**<0.001**
Physical activity score	0.32	0.13–0.52	**<0.001**	0.23	0.08–0.39	**0.003**
NT‐proBNP (ng/L)	−0.19	−0.39 to 0.01	0.065	−0.07	−0.24 to 0.10	0.429
Pulmonary function						
FEV_1_ (l)	0.35	0.16–0.55	**<0.001**	0.22	0.06–0.39	**0.010**
Predicted‐FEV_1_ (%)	0.13	−0.08 to 0.33	0.215			
BOS (yes/no)	−0.51	−1.17 to 0.15	0.128			
Cardiac function						
2D‐LVEF (%)	0.03	−0.17 to 0.24	0.748			
3D‐LVEF (%)	0.08	−0.14 to 0.30	0.449			
GLS (%)	−0.17	−0.37 to 0.04	0.113	−0.18	−0.33 to −0.02	**0.030**
MV_E/A_	0.27	0.08–0.47	**0.007**			
*E*/*e*'	−0.25	−0.44 to −0.05	**0.016**	0.03	−0.13 to 0.20	0.704
RV‐s' (cm/s)	0.13	−0.07 to 0.34	0.199			
RVFWS (%)	−0.04	−0.26 to 0.17	0.696			
PASP (mmHg)	−0.01	−0.21 to 0.20	0.946			

*Notes*: All continuous variables (including dependent and independent) are standardized. GLS and RVFWS are fitted as negative values. Significant *p* values (<0.05) are in boldface.

Abbreviations: BOS, bronchiolitis obliterans syndrome; FEV_1_, forced expiratory volume in 1 s; GLS, global longitudinal strain; LVEF, LV ejection fraction; MV_E/A_, ratio of early‐diastolic wave velocity (MV_E_) to late‐diastolic wave velocity (MV_A_); PASP: peak pulmonary artery systolic pressure; RVFWS, right ventricular free‐wall strain. RV‐s', right ventricular systolic myocardial velocity (average of septum and lateral annulus).

ROC analysis showed percent of predicted‐FEV_1_ (but not unadjusted values of FEV_1_) and GLS to have similar and fair abilities to correctly identify survivors with reduced exercise capacity: Percent of predicted‐FEV_1_ (AUC 0.66, 95% CI: 0.55–0.77, *p* = 0.007) and GLS (AUC 0.64, 95% CI: 0.53–0.75, *p* = 0.014). Other parameters of cardiac function failed to significantly predict survivors with reduced exercise capacity. A GLS cut‐off value of −18% gave a sensitivity of 67% and specificity of 62% for correctly identifying survivors with reduced exercise capacity. The prediction abilities increased mildly when tested with the combined probabilities of GLS and percent of predicted‐FEV_1_ (AUC 0.70, 95% CI: 0.59 to 0.81, *p* = 0.001).

### 3.6 N‐terminal pro‐brain‐type natriuretic peptide

Median value of NT‐proBNP was 48 ng/L (range 6–668 ng/L). Elevated NT‐proBNP was found in 14 (14.6%), including nine (64.3%) with impaired LV and/or RV function identified by echocardiography. NT‐proBNP significantly correlated with multiple parameters related to cardiac function, most prominently: 2D‐LVEF (*R* = −0.38, *p* < 0.001), TRP (*R* = 0.37, *p* = 0.002), MAPSE (*R* = −0.36, *p* < 0.001), LV‐s' (*R* = −0.34, *p* = 0.001), 3D‐LVEF (*R* = −0.34, *p* = 0.002, *p* = 0.005), oxygen‐pulse (*R* = −0.30, *p* = 0.003), RV‐GLS (*R* = 0.29, *p* = 0.007), GLS (*R* = 0.26, *p* = 0.011), *E*/*e*' (*R* = 0.26, *p* = 0.012), cardiac output (*R* = −0.24, *p* = 0.022), RV‐s' (*R* = −0.23, *p* = 0.024) and FAC (*R* = −0.23, *p* = 0.024). NT‐proBNP was not significantly (*p* = 0.429) associated with VO_2peak_ in the multivariable regression.

## 4 DISCUSSION

In this cohort, 46% of long‐term survivors of allo‐HSCT who were treated as children, adolescents and young adults (CAYA) were found to have reduced exercise capacity. With modern echocardiographical techniques such as GLS, we showed that LV systolic function was a significant and independent predictor for VO_2peak_ (ml/kg/min). We are not aware of previous literature reporting similar findings in survivors of allo‐HSCT.

Recently, we reported on pulmonary function in relation to cardio‐respiratory fitness in this cohort.^20^ We found that reduced gas diffusion (DL_CO_), physical de‐conditioning (low VO_2peak_ in absence of cardiac (LVEF) or pulmonary limitations) and low 2D‐LVEF as factors associated with reduced percent of predicted‐VO_2peak._ ^20^ This complimentary study differs by describing in detail cardiac function (beyond 2D‐LVEF) in survivors and in those individuals found to have reduced exercise capacity.

Exercise capacity (VO_2peak_) is affected by many non‐cardiac factors that are taken into consideration in calculations of predicted values. In common with previous studies, we found sex, BMI and physical activity to be significantly associated with VO_2peak_. Other potential factors to consider in this cohort are the temporal aspects of age at transplantation and follow‐up time. Treatment at a young age may disrupt growth and development, while the long follow‐up time prolongs de‐conditioning, increases the risk of accumulating cardiovascular risk factors and promotes sedentary lifestyles. However, it should be recognized that this cohort represents survivors that have evaded the most serious short‐term complications and have benefited from long recovery times. Hence, follow‐up regimes in young recipients of allo‐HSCT should encourage healthy lifestyles and include exercise rehabilitation.

A distinct feature of this cohort was the complex and unique set of risk factors for reduced oxygen uptake. Deciphering the effects related to cardiac dysfunction was compounded by co‐existing pulmonary disorders. The results showed cardiac and pulmonary dysfunction to have similar levels of association with reduced exercise capacity. Cardiac dysfunction as an explanatory factor was supported by corresponding reductions in oxygen‐pulse. The presumed causes for cardiac dysfunction are myocardial impairment and reduced contractility induced by anthracyclines, and the secondary effects from cardiovascular risk factors such as increased afterload due to hypertension. Pulmonary dysfunction may have arisen from complications from chemotherapy, and/or the effects of BOS secondary to chronic GVHD. These findings illustrate the importance of considering both heart and lung dysfunction as reasons for exercise intolerance in survivors of allo‐HSCT.

Echocardiography performed at rest is a surrogate measure of actual myocardial function during exercise. However, multiple studies (mostly unrelated to cardiotoxic therapies) have shown relationships between functional capacity and echocardiographical measurements of systolic function,^11^, ^12^, ^31^ diastolic function^32^, ^33^ and RV function.^34^, ^35^ In this cohort, we found multiple and overlapping phenotypes of cardiac dysfunction. Most common (44%) were cases of mild to moderate LVSD that were strongly associated with RVSD.^13^ A categorization of LVSD gave a significant increase in risk (OR 2.5) for reduced exercise capacity. Arguably, clinically relevant reductions in oxygen uptake were reserved to cases with readily identifiable cardiac dysfunction (2D‐LVEF <50%). Comparisons also showed RV function (RV strain) to be lower in survivors with reduced exercise capacity. However, the effects of reduced RV function were conditional to the presence of co‐existing LVSD and were not significant enough to independently predict VO_2peak_ alone. Elevated filling pressures were found in approximately half of the cases with LVSD. The consequences of elevated filling pressures in this study are not entirely known. Parameters of diastolic function (measured at rest) were significantly correlated with VO_2peak_, however were not significant in multivariable regressions. This may be due to interactions with factors of BMI, age and blood pressure.

In similarity with the larger St. Jude Lifetime cohort by Ness et al., that examined long‐term (≥10 years) adult survivors of childhood cancer, we also found GLS to be superior to 2D‐LVEF (and also 3D‐LVEF in our study) for predicting exercise capacity.^12^ A possible explanation for 2D‐LVEF inability to establish associations with VO_2peak_ may in part be due to geometric assumptions used in its calculation. The higher sensitivity of GLS to detect mild reductions due to cardiotoxicity, better reproducibility and lower variability compared to 2D or 3D‐LVEF are other possible reasons.^6^, ^7^, ^8^, ^9^ It is also likely that the level of association between parameters of systolic function and exercise capacity is underestimated in our study due to exclusion of several patients with heart disease for CPET.

This study also included the biomarker NT‐proBNP owing to its moderate ability in predicting VO_2peak_ in patients with heart failure.^36^ Most instances of elevated NT‐proBNP in this cohort were found in survivors with more pronounced cardiac dysfunction. It was notable that NT‐proBNP was within normal limits in many participants with cardiac dysfunction by imaging, and was not associated with VO_2peak_.

CPET is considered the gold standard for assessing functional capacity in patients with heart disease and the assessment of VO_2peak_ provides valuable prognostic information, including all‐cause mortality.^3^, ^4^, ^5^ In childhood survivors of cancer with reduced exercise capacity (VO_2peak_ < 85% of predicted) the hazard rate for death increases by approximately fourfold.^12^ While, a finding of VO_2peak_ > 20 ml/kg/min in patients with heart failure is considered to correspond to better short‐term prognosis.^37^ To our knowledge, there are no other data documenting the prognostic impact of mild or moderate reduction in exercise capacity in long‐term survivors of HSCT. However, the ability to identify cardiac dysfunction as a cause for reduced exercise capacity (even if mild) has obvious medical value and potential prognostic benefits.

A limitation with CPET is its inability to distinguish specific cardiac mechanisms for reduced oxygen uptake. Thus, identifying tools that explain exercise intolerance is important for clinical decision‐making. This is especially relevant in survivors of allo‐HSCT with multiple co‐morbidities and with uncertain origins of functional dyspnea. We recommend GLS in screening of allo‐HSCT survivors to confirm cardiac dysfunction (irrespective of symptoms) and to provide explanation for reduced oxygen uptake. As demonstrated in this study, GLS when measured at rest had superior ability to predict VO_2peak_ compared with NT‐proBNP and other tested echocardiographical parameters. Lower measurement variability for GLS is one possible explanation for this occurrence. Moreover, GLS has been shown to have valuable prognostic capacity.^6^, ^7^, ^10^ Our study was not designed to address mortality, although based on our findings we are supportive. Finally, the use of GLS is endorsed by experts in cardio‐oncology for early detection of cardiotoxicity, which assists in earlier therapeutic interventions to hinder progressive heart disease associated with reductions in LVEF.^28^, ^38^

## 5 CONCLUSION

Reduced exercise capacity in long‐term survivors of allo‐HSCT treated in their youth was found to be associated with left ventricular systolic dysfunction by GLS, impaired pulmonary function by FEV_1_, increased BMI and lower levels of physical activity. In conclusion, we recommend GLS for identifying and monitoring of left ventricular systolic dysfunction, and for providing an explanation for exercise intolerance in recipients of cancer related therapies.

## 6 STRENGTHS AND LIMITATIONS

The strengths of this study are the nationwide inclusion, completeness in the cohort and comprehensive clinical evaluation of the participants. Interpretations from cross‐sectional studies are limited by the absence of longitudinal data and the prognostic significance of findings is unknown. Echocardiography was performed at rest and considerations need to be taken when evaluating correlations with VO_2peak_. For patient safety reasons, several participants with cardiovascular disease were exempted from CPET. This introduced potential selection bias and likely led to an underestimation of the predictive power of echocardiography and NT‐proBNP on exercise capacity.

**Abbreviations**
Allo‐HSCT: allogeneic hematopoietic stem‐cell transplantation
ANOVA/ANCOVA: analysis of variance/covariance
AUC: area under curve
BMI: body mass index
BOS: bronchiolitis obliterans syndrome
CPET: cardio‐pulmonary exercise test
CAYA: children, adolescents and young adults
FAC: fractional area change
FEV_1_
: forced expiratory volume in 1 s
GLS: global longitudinal strain
GVHD: graft versus‐host‐disease (aGVHD: acute, cGVHD: chronic)
HDL: high‐density lipoprotein
HR: heart rate
LV: left ventricular
LV‐*e*': mean myocardial early diastolic velocity
LDL: low‐density lipoprotein
LVEF: left ventricular ejection fraction
LV‐s': mean systolic myocardial velocity (average of septum and lateral annulus)
LVSD: left ventricular systolic dysfunction
MAPSE: mitral annular plane systolic excursion (average of septum and lateral annulus)
MV: mitral valve
MV_A_
: mitral valve late‐diastolic wave velocity
MV_DT_
: mitral valve deceleration‐time
MV_E/A_
: ratio of mitral valve early diastolic wave velocity (MV_E_) to MV_A_
PASP: pulmonary artery systolic pressure
NT‐ProBNP: N‐terminal pro‐b‐type natriuretic peptide
NYHA: New York Heart Association
RER: respiratory exchange ratio
ROC: receiver operating characteristic
RV: right ventricular
RV‐s': right ventricular systolic velocity (average of septum and lateral annulus)
RVFWS: right ventricular free‐wall strain
RV‐GLS: right ventricular global longitudinal strain
RVSD: right ventricular systolic dysfunction
SBP: systolic blood pressure
STE: speckle tracking echocardiography
TAPSE: tricuspid annulus plane systolic excursion
TRP: tricuspid regurgitation pressure
VIF: variance inflation factor

## AUTHOR CONTRIBUTIONS

All stated co‐authors have fulfilled the ICMJE criteria for authorship. Design and administration of the ‘Norwegian allo‐HSCT survivorship study’ was by Ellen Ruud. Svend Aakhus made subsequent protocols for evaluation of cardiac function. Richard John Massey, Ole Henrik Myrdal, Phoi Phoi Diep, and Marta Maria Burman were responsible for data collection. Echocardiograms were performed by Richard John Massey and analyzed by Richard John Massey with guidance from Svend Aakhus. Professional advice of study methodology and interpretation of results was given by Ellen Ruud, Phoi Phoi Diep, Lars Lysgaard Gullestad, Svend Aakhus, and Jan Otto Beitnes. Jan Otto Beitnes gave supervision to study conduct. All authors contributed to reviewing and editing of the manuscript.

## FUNDING INFORMATION

The Norwegian Extra‐foundation and the Norwegian Cancer foundation provided funding for this study. Open access publication is funded by Oslo University, Norway. No funding was received from the private industry.

## ETHICS STATEMENT

The Norwegian Regional Committee for Medical and Health Research Ethics (2014/370) approved this study. Informed consent was obtained from all participants. All authors have read and approved the final manuscript for publication. There are no conflicts of interest and nothing to declare.

## Supporting information


**Table S1** Cardiac function by echocardiography in survivors with normal (VO_2peak_ > 85% of predicted) and mildly reduced exercise capacity (V0_2peak_ between 75% and 85% of predicted) and moderately reduced (VO_2peak_ < 75% of predicted).

## Data Availability

The datasets generated and/or analyzed during the current study are not publicly available due restrictions set by Norwegian Regional Committee for Medical Research Ethics, but are available from the corresponding author on reasonable request.

## References

[1] Passweg JR , Baldomero H , Bader P , et al. Hematopoietic stem cell transplantation in Europe 2014: more than 40000 transplants annually. Bone Marrow Transplant. 2016;51(6):786‐792. 26901709 10.1038/bmt.2016.20 PMC4895175

[2] Gooley TA , Chien JW , Pergam SA , et al. Reduced mortality after allogeneic hematopoietic‐cell transplantation. N Engl J Med. 2010;363(22):2091‐2101. 21105791 10.1056/NEJMoa1004383 PMC3017343

[3] Mancini DM , Eisen H , Kussmaul W , Mull R , Edmunds LH Jr , Wilson JR . Value of peak exercise oxygen consumption for optimal timing of cardiac transplantation in ambulatory patients with heart failure. Circulation. 1991;83(3):778‐786. 1999029 10.1161/01.cir.83.3.778

[4] Myers J , Gullestad L . The role of exercise testing and gas‐exchange measurement in the prognostic assessment of patients with heart failure. Curr Opin Cardiol. 1998;13(3):145‐155. 9649936

[5] Guazzi M , Adams V , Conraads V , et al. EACPR/AHA scientific statement. Clinical recommendations for cardiopulmonary exercise testing data assessment in specific patient populations. Circulation. 2012;126(18):2261‐2274. 22952317 10.1161/CIR.0b013e31826fb946 PMC4777325

[6] Patel AA , Labovitz AJ . Advanced echocardiographic techniques in detection of cardiotoxicity. Curr Treat Options Cardiovasc Med. 2016;18(4):28. 26922568 10.1007/s11936-016-0450-1

[7] Stanton T , Leano R , Marwick TH . Prediction of all‐cause mortality from global longitudinal speckle strain: comparison with ejection fraction and wall motion scoring. Circ Cardiovasc Imaging. 2009;2(5):356‐364. 19808623 10.1161/CIRCIMAGING.109.862334

[8] Thavendiranathan P , Poulin F , Lim KD , Plana JC , Woo A , Marwick TH . Use of myocardial strain imaging by echocardiography for the early detection of cardiotoxicity in patients during and after cancer chemotherapy: a systematic review. J Am Coll Cardiol. 2014;63(25 Pt A):2751‐2768. 24703918 10.1016/j.jacc.2014.01.073

[9] Thavendiranathan P , Grant AD , Negishi T , Plana JC , Popovic ZB , Marwick TH . Reproducibility of echocardiographic techniques for sequential assessment of left ventricular ejection fraction and volumes: application to patients undergoing cancer chemotherapy. J Am Coll Cardiol. 2013;61(1):77‐84. 23199515 10.1016/j.jacc.2012.09.035

[10] Kalam K , Otahal P , Marwick TH . Prognostic implications of global LV dysfunction: a systematic review and meta‐analysis of global longitudinal strain and ejection fraction. Heart. 2014;100(21):1673‐1680. 24860005 10.1136/heartjnl-2014-305538

[11] Hasselberg NE , Haugaa KH , Sarvari SI , et al. Left ventricular global longitudinal strain is associated with exercise capacity in failing hearts with preserved and reduced ejection fraction. Eur Heart J Cardiovasc Imaging. 2015;16(2):217‐224. 25552469 10.1093/ehjci/jeu277 PMC4307775

[12] Ness KK , Plana JC , Joshi VM , et al. Exercise intolerance, mortality, and organ system impairment in adult survivors of childhood cancer. J Clin Oncol. 2020;38(1):29‐42. 31622133 10.1200/JCO.19.01661 PMC7051850

[13] Massey RJ, Diep PP, Ruud E, Burman MM, Kvaslerud AB, Brinch L, et al. Left Ventricular Systolic Function in Long‐Term Survivors of Allogeneic Hematopietic Stem Cell Transplantation. JACC CardioOncol. 2020;2(3):460‐71. doi:10.1016/j.jaccao.2020.06.011 PMC8352258 34396253

[14] The Criteria Committe of the New York Heart Association . Nomenclature and Criteria for Diagnosis of Diseases of the Heart and Great Vessels. 9th ed. Little, Brown and Co.; 1994.

[15] Children's Oncology Group (COG) . Long‐Term Follow‐Up Guidelines for Survivors of Childhood, Adolescent, and Young Adult Cancers, Version 5.0, Section 33: page 40. 2018. Accessed February 2020. www.survivorshipguidelines.org

[16] Glucksberg H , Storb R , Fefer A , et al. Clinical manifestations of graft‐versus‐host disease in human recipients of marrow from HL‐A‐matched sibling donors. Transplantation. 1974;18(4):295‐304. 4153799 10.1097/00007890-197410000-00001

[17] Shulman HM , Cardona DM , Greenson JK , et al. NIH consensus development project on criteria for clinical trials in chronic graft‐versus‐host disease: II. The 2014 pathology working group report. Biol Blood Marrow Transplant. 2015;21(4):589‐603. 25639770 10.1016/j.bbmt.2014.12.031 PMC4359636

[18] Kurtze N , Rangul V , Hustvedt BE . Reliability and validity of the international physical activity questionnaire in the Nord‐Trondelag health study (HUNT) population of men. BMC Med Res Methodol. 2008;8:63. 18844976 10.1186/1471-2288-8-63 PMC2577099

[19] Miller MR , Hankinson J , Brusasco V , et al. Standardisation of spirometry. Eur Respir J. 2005;26(2):319‐338. 16055882 10.1183/09031936.05.00034805

[20] Myrdal OH, Diep PP, Ruud E, Brinch L, Massey RJ, Edvardsen E, et al. Determinants of cardiorespiratory fitness in very long‐term survivors of allogeneic hematopoietic stem cell transplantation: a national cohory study. Support Care Cancer. 2021;29(4):1959‐67. doi:10.1007/s00520-020-05644-1 PMC7892519 32827056

[21] Quanjer PH , Stanojevic S , Cole TJ , et al. Multi‐ethnic reference values for spirometry for the 3‐95‐yr age range: the global lung function 2012 equations. Eur Respir J. 2012;40(6):1324‐1343. 22743675 10.1183/09031936.00080312 PMC3786581

[22] Jagasia MH , Greinix HT , Arora M , et al. National Institutes of Health consensus development project on criteria for clinical trials in chronic graft‐versus‐host disease: I. The 2014 diagnosis and staging working group report. Biol Blood Marrow Transplant. 2015;21(3):389‐401. 25529383 10.1016/j.bbmt.2014.12.001 PMC4329079

[23] Balke B , Ware RW . An experimental study of physical fitness of Air Force personnel. US Armed Forces Med J. 1959;10(6):675‐688. 13659732

[24] Edvardsen E , Hansen BH , Holme IM , Dyrstad SM , Anderssen SA . Reference values for cardiorespiratory response and fitness on the treadmill in a 20‐ to 85‐year‐old population. Chest. 2013;144(1):241‐248. 23287878 10.1378/chest.12-1458

[25] American Thoracic S , American College of Chest P . ATS/ACCP statement on cardiopulmonary exercise testing. Am J Respir Crit Care Med. 2003;167(2):211‐277. 12524257 10.1164/rccm.167.2.211

[26] Lang RM , Badano LP , Mor‐Avi V , et al. Recommendations for cardiac chamber quantification by echocardiography in adults: an update from the American Society of Echocardiography and the European Association of Cardiovascular Imaging. J Am Soc Echocardiogr. 2015;28(1):1‐39. 25559473 10.1016/j.echo.2014.10.003

[27] Nagueh SF , Smiseth OA , Appleton CP , et al. Recommendations for the evaluation of left ventricular diastolic function by echocardiography: an update from the American Society of Echocardiography and the European Association of Cardiovascular Imaging. Eur Heart J Cardiovasc Imaging. 2016;17(12):1321‐1360. 27422899 10.1093/ehjci/jew082

[28] Plana JC , Galderisi M , Barac A , et al. Expert consensus for multimodality imaging evaluation of adult patients during and after cancer therapy: a report from the American Society of Echocardiography and the European Association of Cardiovascular Imaging. Eur Heart J Cardiovasc Imaging. 2014;15(10):1063‐1093. 25239940 10.1093/ehjci/jeu192 PMC4402366

[29] Massey RJ, Diep PP, Burman MM, Kvaslerud AB, Brinch L, Aakhus S, et al. Impaired right ventricular function in long‐term survivors of allogeneic haematopoietic stem‐cell transplantation. Open Heart. 2012;8(2):e001768. doi:10.1136/openhrt-2021-001768 PMC8693167 34933961

[30] Rudski LG , Lai WW , Afilalo J , et al. Guidelines for the echocardiographic assessment of the right heart in adults: a report from the American Society of Echocardiography endorsed by the European Association of Echocardiography, a registered branch of the European Society of Cardiology, and the Canadian Society of Echocardiography. J Am Soc Echocardiogr. 2010;23(7):685‐713. quiz 86‐8. 20620859 10.1016/j.echo.2010.05.010

[31] Murbraech K , Smeland KB , Holte H , et al. Heart failure and asymptomatic left ventricular systolic dysfunction in lymphoma survivors treated with autologous stem‐cell transplantation: a National Cross‐Sectional Study. J Clin Oncol. 2015;33(24):2683‐2691. 26169610 10.1200/JCO.2015.60.8125

[32] Christiansen JR , Kanellopoulos A , Lund MB , et al. Impaired exercise capacity and left ventricular function in long‐term adult survivors of childhood acute lymphoblastic leukemia. Pediatr Blood Cancer. 2015;62(8):1437‐1443. 25832752 10.1002/pbc.25492

[33] Smart N , Haluska B , Leano R , Case C , Mottram PM , Marwick TH . Determinants of functional capacity in patients with chronic heart failure: role of filling pressure and systolic and diastolic function. Am Heart J. 2005;149(1):152‐158. 15660047 10.1016/j.ahj.2004.06.017

[34] Salerno G , D'Andrea A , Bossone E , et al. Association between right ventricular two‐dimensional strain and exercise capacity in patients with either idiopathic or ischemic dilated cardiomyopathy. J Cardiovasc Med (Hagerstown). 2011;12(9):625‐634. 21792023 10.2459/JCM.0b013e328349a268

[35] Murbraech K , Holte E , Broch K , et al. Impaired right ventricular function in long‐term lymphoma survivors. J Am Soc Echocardiogr. 2016;29(6):528‐536. 27038515 10.1016/j.echo.2016.02.014

[36] Felker GM , Whellan D , Kraus WE , et al. N‐terminal pro‐brain natriuretic peptide and exercise capacity in chronic heart failure: data from the heart failure and a controlled trial investigating outcomes of exercise training (HF‐ACTION) study. Am Heart J. 2009;158(4 Suppl):S37‐S44. 19782787 10.1016/j.ahj.2009.07.011 PMC3748954

[37] Arena R , Myers J , Guazzi M . Cardiopulmonary exercise testing is a core assessment for patients with heart failure. Congest Heart Fail. 2011;17(3):115‐119. 21609384 10.1111/j.1751-7133.2011.00216.x

[38] Armenian SH , Lacchetti C , Lenihan D . Prevention and monitoring of cardiac dysfunction in survivors of adult cancers: American Society of Clinical Oncology clinical practice guideline summary. J Oncol Pract. 2017;13(4):270‐275. 27922796 10.1200/JOP.2016.018770

