# Supplementary material for: Reduced exercise capacity is associated with left ventricular systolic dysfunction in long‐term survivors of allogeneic hematopoietic stem‐cell transplantation
Source: J Clin Ultrasound. 2022 Jul 5;51(1):5–15. doi: 10.1002/jcu.23264 (PMC10084426; doi:10.1002/jcu.23264)
Supplement: Supplementary file 1 — Table S1 Cardiac function by echocardiography in survivors with normal (VO2peak > 85% of predicted) and mildly reduced exercise capacity (V02peak between 75% and 85% of predicted) and moderately reduced (VO2peak < 75% of predicted). [file JCU-51-5-s001.docx]

Supplement Table 1: Cardiac function by echocardiography in survivors with normal (V0_2peak_ >85% of predicted) and mildly reduced exercise capacity (V0_2peak_ between 75% to 85% of predicted) and moderately reduced (V0_2peak_ <75% of predicted).

| **Variable** | **All survivors** | | **VO_2peak_ <75%**  **of predicted** | | **VO_2peak_ 75% to 85%**  **of predicted** | | **VO_2peak_ >85%**  **of predicted** | | | **p-value *** | | **Adjusted**  **p-value †** |
| --- | --- | --- | --- | --- | --- | --- | --- | --- | --- | --- | --- | --- |
| Number | 96 | | 20 | | 23 | | 53 | | | **-** | | **-** |
| VO_2peak_ (ml/kg/min) (range) | 36.2 ± 7.7  (19 - 54) | | 28.0 ± 5.5  (19 - 39) | | 36.0 ± 6.4  (26 - 44) | | 39.4 ± 6.6  (28 - 54) | | | **<0.001 a, b** | | **<0.001 a, b, c** |
| Peak heart rate (bpm) | 181 ± 15 | | 182 ± 16 | | 185 ± 15 | | 179 ± 15 | | | 0.359 | | **-** |
| Oxygen pulse (ml/beat) | 14.1 ± 3.8 | | 12.1 ± 3.0 | | 13.6 ± 2.6 | | 15.1 ± 4.1 | | | **0.007 a** | | **<0.001 a, c** |
| FEV_1_ (l/sec) | 3.3 ± 0.8 | | 2.9 ± 0.9 | | 3.3 ± 0.7 | | 3.4 ± 0.8 | | | 0.088 | | **0.007 a** |
| Anthracycline exposure | 43 (44.8) | | 12 (60.0) | | 10 (43.5) | | 21 (39.2) | | | 0.292 | | **-** |
| Anthracycline dosage (mg/m^2^) | 0 (0, 219) | | 75 (0, 405) | | 0 (0, 205) | | 0 (0, 207) | | | 0.276 | | **-** |
| NT-proBNP (ng/l) | 48 (22,83) | | 71 (21, 142) | | 45 (14, 82) | | 47 (27, 82) | | | 0.300 | | **-** |
| NYHA class ≥II | 25 (26.0) | | 12 (60.0) | | 6 (26.1) | | 7 (13.2) | | | **<0.001 a, b** | | **-** |
| Physical activity score | 3.8 (1.5, 5.0) | | 1.5 (0.2, 3.8) | | 3.8 (1.5, 5.6) | | 3.8 (1.9, 6.6) | | | **0.009 a, b** | | - |
| **SYSTOLIC FUNCTION** |  | |  | |  | |  | | |  | |  |
| Cardiac Output (l/min) | 4.77 ± 1.13 (n=95) | | 4.73 ± 1.10 | | 4.97 ± 1.13 | | 4.70 ± 1.16 (n= 52) | | | 0.637 | | 0.082 |
| Fraction Shortening (%) | 31.0 ± 5.3 | | 28.5 ± 5.6 | | 31.9 ± 5.2 | | 31.5 ± 5.1 | | | 0.058 | | 0.054 |
| 2D-LVEF (%) | 55.4 ± 5.9 | | 52.3 ± 6.6 | | 56.1 ± 5.5 | | 56.3 ± 5.5 | | | **0.025 a** | | **0.010 a** |
| 3D-LVEF (%) | 54.1 ± 4.9 (n= 83) | | 50.5 ± 6.9 (n= 13) | | 54.3 ± 3.7 (n= 21) | | 55.0 ± 4.4 (n= 49) | | | **0.013 a** | | **0.015 a** |
| GLS (%) | -17.6 ± 2.0 (n= 94) | | -16.4 ± 2.1 (n= 19) | | -17.7 ± 1.7 | | -18.0 ±1.9 (n= 52) | | | **0.006 a** | | **0.010 a** |
| MAPSE (mm) | 13.1 ± 2.0 | | 12.2 ± 2.1 | | 13.4 ± 1.5 | | 13.2 ± 2.1 | | | 0.078 | | **0.025 a** |
| LV-s' (cm/s) | 8.1 ± 1.7 | | 8.1 ± 1.4 | | 8.4 ± 1.6 | | 8.0 ±1.8 | | | 0.525 | | 0.540 |
| **DIASTOLIC FUNCTION** |  | |  | |  | |  | | |  | |  |
| MV_DT_ (ms) | 160 ± 39 | | 168 ± 38 | | 154 ± 52 | | 160 ± 33 | | | 0.465 | | 0.147 |
| MV_E/A_ ratio | 1.6 ± 0.8 | | 1.5 ± 0.5 | | 1.7 ± 0.8 | | 1.7 ± 0.9 | | | 0.475 | | 0.272 |
| e' (cm/s) | 11.1 ± 3.0 | | 11.0 ± 3.0 | | 11.4 ± 3.0 | | 11.0 ± 3.1 | | | 0.822 | | 0.454 |
| E/e' | 6.4 ± 2.1 | | 6.0 ± 1.5 | | 6.8 ± 1.9 | | 6.5 ± 2.4 | | | 0.454 | | 0.298 |
| **RIGHT VENTRICULAR FUNCTION** | |  | |  | |  | | |  | |  | |
| FAC (%) | 41.1 ± 5.3 | | 40.0 ± 6.6 | | 42.8 ± 4.5 | | | 40.9 ± 5.0 | | 0.197 | | 0.290 |
| RVFWS (%) | -27.0 ± 4.4 (n= 89) | | -25.3 ± 4.6 (n= 17) | | -27.4 ± 3.6 (n= 21) | | | -27.4 ± 4.5 (n= 51) | | 0.199 | | **0.045 a** |
| RV-GLS (%) | -21.8 ± 3.2 (n= 89) | | -20.5 ± 3.2 (n= 17) | | -22.1 ± 2.9 (n= 21) | | | -22.1 ± 3.3 (n= 51) | | 0.184 | | **0.030 a** |
| TAPSE (mm) | 20.8 ± 3.7 | | 19.8 ± 3.8 | | 21.6 ± 3.2 | | | 20.9 ± 3.8 | | 0.274 | | 0.243 |
| RV-s' (cm/s) | 11.1 ± 2.2 | | 10.3 ± 2.6 | | 12.2 ± 1.6 | | | 10.9 ± 2.2 (n= 52) | | **0.015 b** | | **0.007 b** |
| TRP (mmHg) | 17.7 ± 3.4 (n= 72) | | 16.0 ± 1.9 (n= 13) | | 18.7 ± 3.3 (n= 18) | | | 17.8 ± 3.6 (n= 41) | | 0.073 | | 0.089 |
| Values presented as n (% in group), mean ± SD or median (25^th^, 75^th^ percentiles). Significant p-values (<0.05) are in boldface. **^*^** ANOVA, Kruskal-Wallis test, Chi-square test. ^†^ ANCOVA with covariates of age at examination, BMI, heart rate at echocardiography and systolic blood pressure at echocardiography.  a: Bonferroni correction: Significant difference (p<0.05) between (<75% of predicted-VO_2peak_) with normal (>85% of predicted-VO_2peak_).  b: Bonferroni correction: Significant difference (p<0.05) between (<75% of predicted-VO_2peak_) and (75% to 85% of predicted-VO_2peak_).  c: Bonferroni correction: Significant difference (p<0.05) between (75% to 85% of predicted-VO_2peak_) and normal (>85% of predicted-VO_2peak_).  **Abbreviations:** e': Mean myocardial early-diastolic velocity, E/e': MV_E_: e' ratio, FAC: Fractional area shortening. GLS: Global longitudinal strain, LV: Left ventricular, LV-s': LV systolic myocardial velocity (average of septum and lateral annulus), LVEF: LV ejection fraction, MAPSE: Mitral annular plane systolic excursion (average of septum and lateral annulus), MV: Mitral valve, MV_DT_: MV deceleration-time, MV_E/A_: Ratio of MV early-diastolic wave velocity (MV_E_) to MV late-diastolic wave velocity (MV_A_), NT-ProBNP: N-terminal pro-b-type natriuretic peptide, RV: Right Ventricular, RV-s': RV systolic velocity (average of septum and lateral annulus), RVFWS: RV-free-wall strain, RV-GLS: RV-global longitudinal strain, TAPSE: Tricuspid annulus plane systolic excursion and TRP: Tricuspid regurgitation pressure. | | | | | | | | | | | | |
